# The cerebral cavernous malformations proteins

**DOI:** 10.18632/oncotarget.5443

**Published:** 2015-08-31

**Authors:** Xiaofeng Li, Oriana S. Fisher, Titus J. Boggon

**Affiliations:** Departments of Pharmacology and Molecular Biophysics and Biochemistry, Yale University School of Medicine, New Haven, CT, USA

**Keywords:** signal transduction, CCM disease

Inherited mutations in three genes lead to the familial form of Cerebral Cavernous Malformations (CCM). These vascular dysplasias most commonly occur in the brain, and manifest as dilated, mulberry-shaped lesions with a single endothelial layer. The consequences of these lesions can be leakage and sequelae such as focal neurological deficits, epilepsy, or hemorrhagic stroke. Until recently, however, the molecular basis for the acquisition of CCM disease was unknown. The three genes associated with CCM disease encode the proteins KRIT1/CCM1 (Krev interaction trapped 1/cerebral cavernous malformations 1), CCM2/malcavernin/OSM (cerebral cavernous malformations 2, osmosensing scaffold for MEKK3), and CCM3/PDCD10 (cerebral cavernous malformations 3/programmed cell death 10). Maintenance of a normal vasculature requires expression of all three proteins. Almost all of the discovered mutations in these genes result in truncations of their protein products, although some rare missense mutations have been found to encode misfolded protein [1]. The three proteins implicated in the disease were predicted to be distinct from one another in structure, but their molecular level architecture and many details of their normal function and protein-protein interaction networks were unknown. Therefore, to better understand the signaling processes that are affected by CCM disease it was necessary to address these questions from the ground up, starting at the atomic level [2].

The formation of a ‘CCM complex’ between KRIT1, CCM2 and CCM3 was previously suggested, but without targeted disruption of the interactions and selective probing of the functional consequences of disruption, the specific role(s) of heterotrimerization have been hard to define. We tackled these questions using a structure-directed approach. Our studies revealed the molecular basis of a preferred interaction site between KRIT1 and CCM2 [1] and the molecular basis for the interaction of CCM2 with CCM3 [3], thus providing the atomic-level framework for the CCM complex. When we crystallized CCM3, we found that it is of an unexpected fold encompassing two domains [4], the C-terminal of which directly interacts with a conserved motif in CCM2 [3]. Targeted disruption of the interaction between CCM2 and CCM3 has a number of functional consequences. CCM2 and CCM3 reciprocally stabilize one another; knockdown of either CCM2 or CCM3 results in severely reduced stability of the other protein. Importantly, the decreased stability can be rescued by re-expression of the wild-type protein but not protein that has been mutated at the binding site. Loss of either expressed protein also deleteriously impacts proliferation and network formation in endothelial cells. Interestingly, CCM3 expression can rescue proliferation in CCM2 depleted cells, but CCM2 cannot rescue expression in CCM3 depleted cells and the interaction surface between CCM2 and CCM3 must be preserved to rescue proliferation. Conversely, CCM2 is better able to rescue endothelial network formation than CCM3. An important role of the CCM complex in endothelial cells therefore seems to be stabilization of the CCM proteins, allowing them to achieve their overlapping but distinct roles.

The CCM proteins also interact with other signaling proteins. We, and others, have shown the molecular basis for interactions of KRIT1 with the Rap1 small GTPase [5] and with the suppressor of integrin activation, ICAP1 [6]. We have also very recently discovered how CCM2 interacts with the MAP kinase kinase kinase, MEKK3. A previously uncharacterized N-terminal helical region of MEKK3 directly binds the C-terminal HHD (harmonin homology domain) of CCM2 [7]. Targeted disruption of this interaction was not observed to have an impact on MEKK3 catalytic activity, but did alter MEKK3 sub-cellular localization. Disruption of the CCM2:MEKK3 interaction also increases Rho/ROCK signaling, potentially implying that this upregulation may be related to the ability of CCM2 and MEKK3 to interact. *In vivo*, targeted disruption of the CCM2:MEKK3 interaction increased the permeability of the neurovasculature. This study therefore provides a link between CCM2, MEKK3, and the dysregulation of Rho/ROCK signaling that has previously been observed in CCM disease.

Taken together, our recent studies, and those from other groups not mentioned here due to space and formatting constraints, suggest that the proteins of the CCM complex not only require one another for reciprocal stabilization, but also act as a platform for signal transduction. A more intricate understanding of how the CCM complex signaling platform is formed, how its formation is regulated, and how it interacts with binding partners are clearly required. Nonetheless, the molecular level reasons why CCM disease is so distinctly associated with loss of KRIT1, CCM2 and CCM3 are now becoming clearer.
